# Expression profiling of HSP 70 and interleukins 2, 6 and 12 genes of Barki sheep during summer and winter seasons in two different locations

**DOI:** 10.1007/s00484-022-02339-6

**Published:** 2022-07-26

**Authors:** Raed A. Abu Rawash, Mahmoud A. Sharaby, Gamal El-Din A. Hassan, Alaa E. Elkomy, Elsayed E. Hafez, Salma H. Abu Hafsa, Mohamed M. I. Salem

**Affiliations:** 1 Department of Animal and Fish Production, Faculty of Agriculture, University of Alexandria, Alexandria, 21545 Egypt; 2 Department of Livestock Research, Arid Lands Cultivation Research Institute, City for Scientific Research and Technology Applications, New Borg El-Arab, Alexandria, 21934 Egypt; 3 Faculty of Desert and Environmental Agriculture, Matrouh University, Matrouh, Egypt; 4 Plant Protection and Biomolecular Diagnosis Department, City of Scientific Research and Technology Applications, Arid Lands Cultivation Research Institute, New Borg El-Arab City, Alexandria, 21934 Egypt

**Keywords:** Heat stress, HSP 70, Interleukin, Real-time PCR, Sheep, Thermal stress

## Abstract

The objectives of this research were to contrast the expression values of heat shock protein (HSP70) and interleukins 2, 6 and 12 (IL 2, IL 6 and IL 12) genes in summer and winter in two different locations in Egypt (Alexandria zone and Matrouh zone) to deduce changes in thermo-physiological traits and biochemical blood metabolites of Barki sheep. A total of 50 ewes (20 in Alexandria and 30 in Matrouh) were individually blood sampled to determine plasma total protein (TP), Albumin, Globulin and Glucose constituents and T3, T4 and cortisol hormones. The thermo-physiological parameters of rectal temperature (RT, °C), skin temperature (ST, °C), Wool temperature (WT, °C), respiration rate (RR, breaths/min) and pulse rate (PR, beats/min) were measured for each ewe. Expressions of IL 2, IL 6, IL 12 and HSP 70 in summer and winter were analyzed along with thermo-physiological parameters and blood biochemical metabolites. In both locations, THI had significant effects on ST, WT, PR and RR, but not significant on RT. However, it had no significant effects on blood plasma metabolites and hormonal concentrations in the two locations in summer and winter. In Alexandria location, THI had negative significant effect on the expressions of IL-2 and IL-6 but positively affected on HSP70 genes in winter, while the expression of IL-12 gene was not affected by seasons, whereas in Matrouh zone, the effects of THI on the expressions of all tolerance genes were not significant. The results of the current study suggest that IL-2, IL-6 and HSP70 genes could be used as molecular markers for heat/cold stress.

## Introduction

The conditions under which an animal is exposed to sudden changes in the ambient meteorological elements that cause its inability to cope with the endemic environment or failure to achieve prosperity according to its genetic potential are known as climatic stress (Dobson and Smith 2000; Sunil Kumar et al. 2011). In addition to climatic stress, animals could be subjected to other types of stressors such as production, transportation and immune stresses (Mirkena et al., 2010). The ability of an animal to respond to a particular stressor depends on history of previous exposure to adverse circumstances, genetic make-up, age, season and physiological status (Blecha et al. 1983; Mason et al. 1991). The recent phenomenon of global warming causes heat stress to become one of the principal factors that imposes negative impacts on production and reproduction in farm animals (Silanikove et al. 1987). Variation in ambient temperature either above or below the upper or lower critical temperature leads to a condition known as the thermal stress (Pandey et al. 2014). Thermal stress may compromise reproductive efficiency of farm animals in both sexes and hence affect negatively milk, meat, wool production, feed intake, growth rate, immunity and cause changes in blood constituents and most of the biological pathways (Maibam et al. 2018; Skibiel et al. 2018; Becker et al. 2020). To overcome the adverse effect of heat stress caused by global warming, adaptation to hot climate practiced on livestock species became highly imperative in order to increase the adaptive capacity of animals. This involves different physiological pathways, biochemical routes, compositional alterations and hormonal changes to help animals to survive and produce under prevailing climatic conditions (St Pierre et al. 2003). Tolerance or susceptibility to thermal stress varies among animals, but can be explored at the DNA level to identify superior animals that carry genes of adaptability traits, in order to be used in selection programs to improve animal tolerance to heat stress (Singh et al. 2017).

Heat shock proteins (HSP) are a family of proteins that are produced by cells in response to exposure to stressful conditions (Park et al. 2007; Singh et al. 2017). They play essential roles in the cellular homeostasis. HSPs have been classified based on their molecular weights to HSP90 (85–90 kDa), HSP70 (68–73 kDa), HSP60, HSP47, and small HSPs (12–43 kDa) (Park et al. 2007; Singh et al. 2017). One of the most abundant and the best characterized is the HSP70 (Banerjee et al. 2014). These proteins are induced by stress and play essential roles in environmental stress tolerance and thermal adaptation (Banerjee et al. 2014; Garbuz, and Evgen’ev 2016; Singh et al. 2017). Moreover, Interleukin (IL) are a group of cytokines formed of secreted proteins and signal molecules that were first seen to be expressed by leukocytes as IL2, IL6, and IL12 (Bharati et al. 2017). They play a significant role in promoting the immune system and establishing the balance between humoral Th2 and cell mediated Th1 responsiveness (Bharati et al. 2017). In order to determine superior animals that carry the genes of adaptability traits, the current study performed an expression analysis for HSP70, IL2, IL6 and IL12 genes in Barki sheep in summer and winter seasons in two distinct locations (Alexandria zone vs Matrouh zone) in relation to changes in thermo-physiological parameters and biochemical blood components under heat stress and thermoneutral conditions.

## Material and methods

### Animals and locations

The studied animals belonged to Barki sheep breed. The origin of Barki is North Africa in the coastal Mediterranean zone, and characterized by its medium size, light colour and well adapted to survival in the hot arid environment (Abdel–Moneim et al. 2009; Abousoliman et al. 2020), which help to increase heat loss by evaporation while absorbing less heat (Berihulay et al. 2019).

Data were collected from The Sustainable Development Center for Matrouh Resources—Desert Research Center, Matrouh (31° 21′ 11ʺ N, 27° 11′ 05ʺ E) and Animal Production Research Station- Faculty of Agriculture—Alexandria University – Alexandria (31° 12′ 43ʺ N, 29° 59′ 02ʺ E). A total of 50 Barki ewes (30 in Matrouh and 20 in Alexandria) ageing 3–4 years were used in this study. The experiments were carried out in two distinct time spans coinciding with the coldest and hottest periods of year viz. mid-winter (January) and mid-summer (August). The animals were raised under similar management conditions and were monitored throughout the experimental period.

### Blood samples and biochemical parameters

Two blood samples each of 5 ml were collected from each ewe (The samples were taken in the mid of August and mid of January), via jugular vein puncture into a heparinized vials treated with 0.5 ml of 2.75% EDTA (Pspark, UK), as anticoagulant and were transferred immediately to the manipulation laboratory in an ice box for biological and molecular tests. Plasma was analyzed to determine the total protein (TP), Albumin, Globulin and Glucose calorimetrically by Hitachi 901 spectrophotometer and using STANBIO commercial kits. Blood cortisol, triiodothyronine (T3) and thyroxine (T4) concentrations were determined by ELISA method using commercial kits (cortisol, T3 and T4 ELISA monobind Inc., Lake Forest, CA, USA).

### Physiological parameters

At the day of blood sampling, the thermo-physiological parameters of rectal temperature (RT, °C), skin temperature (ST, °C), wool temperature (WT, °C), respiratory rate (RR, breaths/min) and pulse rate (PR, beats/min) were collected from individual ewes early in the morning (7:00–8:00 am) before access to feed and in the afternoon (2:00–3:00 pm).

### Temperature-humidity index (THI)

Data on meteorological variables, ambient temperature (AT) and relative humidity (RH) were obtained mid of August when AT is usually the highest and mid of January when it is the lowest to represent summer and winter seasons. THI was calculated according to the formula: THI = 0.8 × AT + [(RH % ÷ 100) × (AT—14.4)] + 46.4 (Kendall and Webster 2009). The obtained values of THI in Alexandria and Matrouh areas were 81.73 and 80.72 in summer, respectively, while the corresponding values in winter were 60.79 and 65.20, indicating that the experimental animals were under thermoneutral conditions in winter, but suffered from heat stress in summer.

### Total RNA extraction and cDNA preparation

Total RNA was extracted from the blood tissue using total RNA Extraction kit (Total RNA Purification Mini Spin Kit), following the standard protocol of the manufacturer. Quality and quantity of RNA samples were determined by BioDrop (BioDrop µLite, UK). RNA samples giving an optical density (O.D) between 1.8 and 2.0 should contain pure RNA without protein contamination, therefore were utilized in further analysis. Total isolated RNA was treated with DNase I using RNeasy Mini Kit and integrity was determined by 1.5% denaturation agarose gel electrophoresis prior to cDNA synthesis. Total RNA was converted to cDNA using ProtoScript First Strand cDNA synthesis kit (Biolab, UK) following the manufacturer protocol. The prepared cDNA was analyzed using PCR and stored at − 80 °C until further use for qRT-PCR technique.

### Quantitative real-time PCR (qRT-PCR)

Real time-PCR was performed to amplify target and reference genes on Light Cycler 480 (Roche) with Eva Green q.PCR reaction kit (Qarta). It was carried out using the SYBR green following manufacturer instructions. The primers were designed using primer 3 software from NCBI database (Table 1) and the Ensembl (EMBL-EBL Wellcome Trust Sanger Institute, Cambridegeshire, UK). The real-time PCR program was as follows: initial denaturation at 95 °C for 15 min; 40 cycles of 94 °C for 15 s; annealing at 56 °C for 30 s and extension at 72 °C for 30 s. Expression of GAPDH was taken as an endogenous reference (Migaud et al. 2005). Relative quantification of target genes was done by 2^ ^(−ΔΔCt)^ method (Livak and Schmittgen 2001).

**Table 1:** Primer sequence for quantitative real time PCR

Genes	Primers sequences	Product size (bp)	NCBI reference seq
IL-2	F 5′ -CCTGAGCAGGATGGAGAATTACA -3	141	NM_008366.3
	R 5′-TCCAGAACATGCCGCAGAG -3′		
IL-6	F 5′- TCCAGTTGCCTTCTTGGGAC -3′	140	NM_031168.1
	R 5′-GTGTAATTAAGCCTCCGACTTG -3′		
IL-12	F 5′-GGAAGCACGGCAGCAGAATA-3′	179	NM_001303244
	R 5′-AACTTGAGGGAGAAGTAGGAATGG -3′		
HSP70	F 5′-CAGATGAGGCCGTGGCTTAT-3′	101	EH_038080.1
	R 5′-GGGAGTCACATCCAACAGCAA-3′		

*HSP 70*, Heat shock protein 70, IL-2, IL-6, IL-12: Interleukins 2, 6 and 12

### Statistical analysis

Generalized linear model (GLM) was utilized to test the effect of locations (Alexandria city vs Matrouh city), season (summer vs winter) using SAS software (SAS, 2012) according to the following model:

Y_ijk_ = µ + L_i_ + S_j_ + e_ijk_ where,

Y_ijk_: either gene expression profile, biochemical blood components parameters or physiological parameters; µ: an underlying constant specific to each trait; L_i_: the effect of i^th^ location (i = 1 and 2); S_j_: the effect of j^th^ season (j = 1 and 2) and e_ijk_: random errors assumed to be independent normally. Results were presented as means ± SE of a minimum of two independent replicates. Duncan’s multiple range test checked the significance (*P* < 0.05) of the differences between group means (Table 2).

**Table 2:** Seasonal differences in expressions of tolerance genes (Means ± SE) of Barki sheep in Alexandria and Matrouh areas

Item	City	Winter	Summer	*P*-value
IL-2	Alexandria	18.99^b^ ± 0.39	27.98^a^ ± 0.06	0.0001
	Matrouh	29.90 ^NS^ ± 0.20	29.39 ^NS^ ± 0.18	0.09
IL-6	Alexandria	19.08^b^ ± 0.23	28.15^a^ ± 0.07	0.0001
	Matrouh	28.33 ^NS^ ± 0.11	28.29 ^NS^ ± 0.04	0.74
IL-12	Alexandria	9.73^NS^ ± 0.82	10.62^NS^ ± 2.33	0.72
	Matrouh	13.46 ^NS^ ± 3.32	14.58 ^NS^ ± 3.24	0.81
HSP 70	Alexandria	15.24^a^ ± 3.25	7.29^b^ ± 0.09	0.03
	Matrouh	14.97 ^NS^ ± 3.22	15.84 ^NS^ ± 2.97	0.85

HSP 70: Heat shock protein, IL-2, IL-6, IL-12: Interleukins 2, 6 and 12

^a^^,^^b^ Values within a row with different superscripts differ at *P* < 0.05, *NS*, not significant

## Results

Differences between winter and summer seasons for thermo-physiological parameters, blood plasma metabolites and average expression of tolerance genes underlying the ability of Barki ewes to withstand meteorological changes in Alexandria and Matrouh areas are presented in Tables 3 and 4. Changes in THI values from winter to summer had no effect on RT in Alexandria and Matrouh areas. RT of ewes in experimental flocks were consistent at about 39 °C with a range of differences not exceeding 0.1 °C between seasons and between locations. However, ST, WT, PR and RR but not RT were different between seasons in both locations. These thermo-physiological parameters were higher in summer heat stressed ewes compared to those under winter thermoneutral conditions. Seasonal effects on blood plasma metabolites and hormonal concentrations were not significant in both experimental locations. Seasonal effects on expressions of HSP 70, IL-2 and IL-6 but not on IL-12 genes were significant in Alexandria area, showing upregulation in summer compared to winter. While the upregulation of HSP 70 gene expression was detected in Alexandria in winter rather than in summer. In Matrouh area, however, none of the tolerance genes showed significant changes in expression by season but were arbitrarily upregulated in either season.

**Table 3:** Seasonal differences in thermo physiological parameters (Means ± SE) of Barki sheep in Alexandria and Matrouh areas

Item	City	Winter	Summer	*P*-value
RT (°C)	Alexandria	39.21^NS^ ± 0.07	39.28^NS^ ± 0.05	0.30
	Matrouh	39.40 ^NS^ ± 0.07	39.30 ^NS^ ± 0.05	0.27
ST (°C)	Alexandria	29.75^b^ ± 0.25	35.62^a^ ± 0.17	0.0001
	Matrouh	32.77^b^ ± 0.23	36.59^a^ ± 0.14	0.0001
WT (°C)	Alexandria	17.56^b^ ± 0.10	32.30^a^ ± 0.13	0.0001
	Matrouh	22.22^b^ ± 0.15	33.22^a^ ± 0.11	0.0001
PR (pulse/min)	Alexandria	82.16^b^ ± 1.85	87.16^a^ ± 1.10	0.03
	Matrouh	78.364^b^ ± 1.29	88.750^a^ ± 1.61	0.0001
RR (breath/min)	Alexandria	35.00^b^ ± 0.62	69.83^a^ ± 0.96	0.0001
	Matrouh	40.045^b^ ± 1.83	50.57^a^ ± 1.69	0.0001

*RT*, rectal temperature, *ST*, skin temperature, *WT*, wool temperature, *PR*, pulse rate, *RR*, respiration rate

^a^^,^^b^ Values within a row with different superscripts are significantly different at *P* < 0.05, *NS*, not significant

**Table 4:** Seasonal differences in thermo physiological parameters (Means ± SE) of Barki sheep in Alexandria and Matrouh areas

Item	City	Winter	Summer	*P*-value
Total protein	Alexandria	7.64^NS^ ± 0.19	7.26^NS^ ± 0.28	0.291
	Matrouh	7.83 ^NS^ ± 0.17	7.60 ^NS^ ± 0.19	0.369
Albumin	Alexandria	2.66^NS^ ± 0.09	2.49^NS^ ± 0.07	0.141
	Matrouh	2.66 ^NS^ ± 0.08	2.79^NS^ ± 0.03	0.125
Globulin	Alexandria	5.01^NS^ ± 0.17	4.86^NS^ ± 0.26	0.671
	Matrouh	5.29 ^NS^ ± 0.24	4.75 ^NS^ ± 0.15	0.068
Glucose	Alexandria	73.05^NS^ ± 4.99	73.86^NS^ ± 2.89	0.885
	Matrouh	95.67 ^NS^ ± 6.41	80.67 ^NS^ ± 9.47	0.204
T3	Alexandria	1.52 ^NS^ ± 0.12	1.72 ^NS^ ± 0.11	0.283
	Matrouh	1.33 ^NS^ ± 0.15	1.15 ^NS^ ± 0.08	0.233
T4	Alexandria	10.24 ^NS^ ± 0.43	10.72 ^NS^ ± 0.32	0.402
	Matrouh	9.64 ^NS^ ± 0.34	9.21 ^NS^ ± 0.56	0.523
Cortisol	Alexandria	23.80 ^NS^ ± 1.10	21.28 ^NS^ ± 2.09	0.317
	Matrouh	28.82 ^NS^ ± 1.47	26.72 ^NS^ ± 3.22	0.570

*NS*, not significant; *T3*, triiodothyronine; *T4*, thyroxine

## Discussion

### Physiological parameters

Sheep are homoeothermic animals. They maintain body temperature balance by dissipation of the excessive heat outside their body mass through number of biological mechanisms. These biological mechanisms are increasing respiration rate, panting and flushing heat from the body surface to the ambient environment through the skin by conduction, convection, radiation or evaporation (El-Zeiny 2011; Berihulay et al. 2019). Thus, RT is used as an indicator for animal core heat and increases only when body fails to maintain heat balance (Berihulay et al. 2019). Therefore, the consistency of ewes RT in the current study in both locations confirmed the success of these animals to preserve body temperature at the normal level regardless of the increase in THI values above 80 especially in summer in both locations. However, the increase of thermo-physiological parameters of ST, WT, PR and RR in summer when THI increases above 80 verified the commencement of the physiological activities to get rid of the excessive heat. These parameters exhibit an immediate response to the climatic stress and, therefore, provide an evidence for the optimum level of comfort/discomfort level to the animal.

The ability of an animal to withstand the discomfortable thermal conditions is determined by the observed changes in the physiological parameters. Al-Haidary et al. (2012) obtained high average ST value of 38.13 °C in Najdi sheep in the hot Saudi Arabian summer season and Rathwa et al. (2017) found significant increase in ST of Indian sheep from 35.5 °C in winter to 37.8 °C in summer. The high ambient air temperature causes increase of ST as a result of redistribution of blood flow towards the body surface and therefore increase the skin blood flow to transfer heat outside the body ending with regulation of heat between body core and skin (Indu et al. 2014; Marai 2007).

The cardio-respiratory system is influenced by season, day timing, ambient temperature and relative humidity. The first mechanism taken by an animal subjected to heat stress is the increment in RR, which causes loss of heat through evaporation (Renaudeau et al. 2012). Furthermore, Ribeiro et al. (2014) demonstrated that stressed animals utilize respiratory mechanism to avoid increase in RT and maintain homeostasis. Heat stress eventually leads to dissipation of moisture by evaporation from the respiratory tract to maintain thermal balance. These mechanisms are very crucial in preventing hypothermia. Furthermore, Nienaber et al. (2007) revealed that the animal tries to maintain homeostasis by wasting heat load from the body.

In accordance with the current results, Rathwa et al. (2017) reported an increase in RR from 40 breaths/min in winter to 108 in summer and Okoruwa (2014) obtained an increase in RR of African black dwarf goats from 16.04 breaths/min under thermoneutral conditions to 23.01 breaths/min. Moreover, Adedeji (2012) found significant increase in RR of black West African Dwarf Goats compared to white goats from 60.4 breaths/min for to 65.58 breaths/min, which confirmed that black goats are less tolerant to the ambient environmental temperature.

The genetic make-up of sheep influences their response to heat stress measured by change in RR. Joy et al. (2020) reported that Dorper lambs showed less increase in RR than Merino second cross lambs (Poll Dorset × Merino/Border Leicester). Also, variation in breed response to heat stress in goats was apparent (Rout et al. 2018). Comparison between RR of Jamunapari and Barbari goats indicated that RR of the two breeds were 37 and 45 under stress while were 30 and 33 under thermoneutral conditions.

In general, exposure of animals to heat stress causes alterations in circadian rhythm of the cardiac functions including increasing PR which will increase the blood flow from the core to the peripheral of the body. Consequently, previous changes cause higher heat loss by conduction, convection, radiation and water loss by diffusion through the skin (Marai et al. 2007). The increase in PR was found to be positively correlated with RR (Popoola et al. 2014). The capacity of the animal to accommodate the cardiac function alteration related to tolerance to thermal stress was found to be influenced by the genetic make-up and breed differences. For example, coat color of African dwarf goats had a direct effect on PR. During heat stress, PR of black goats counted 83.57 beats/min while white goats recorded 74.97 beats/min (Adedeji 2012). Also, Rout et al. (2018) found that variation in PR of Jamunapari and Barbari goat breeds were significantly high in peak heat stress period recording 111 and 125 beats/min in hot season and 99 vs 108 beats/min under thermoneutral conditions in the two breeds respectively.

### Blood analysis

The non-significant effects of THI values in summer and winter seasons on blood biochemical components in either location indicated that Barki sheep breed succeeded to express high capability to maintain the body biological functions at normal levels. This may be associated with the breed competence to regulate body temperature and maintain it at the normal level to reduce the adverse effect of heat stress of summer by increasing PR, RR, ST and WT (Nienaber et al. 2007; Renaudeau et al. 2012**;** da Silva et al. 2017).

### Relative expression of HSP 70, IL 2, 6 and IL12 genes

In both experimental zones, Barki sheep were under heat stress in summer. The non-significant differences between all expressions of tolerance genes in winter and summer of Matrouh area associated with significant differences for the same genes except IL 12 indicated different response of animals to seasonal variations probably due to different ambient climatic conditions between locations. The upregulation of IL 2 and IL 6 in summer concomitant with upregulation of HSP70 in winter indicated different mode of action for the two types of genes. In Alexandria, HSP70 can be used as a molecular marker for cold adaptation, while IL 2 and IL 6 genes can be used for the same purpose in summer. IL12 has no impact on climatic conditions but more research may be needed to explore other possible roles of the gene.

Similar results by Bharati et al. (2017) illustrated the putative role of IL2 and IL6 as tolerance genes against heat stress; performing the mRNA expression analysis during short- and long-term heat stress acclimation in Tharparkar cattle showed significant increase in IL2 and IL6 expressions after exposure of animals to temperature of 42 °C for 6 h daily for a long period of 23 days. IL2 and IL6 could possibly play a key role to elicit the immune response to ameliorate the thermal insults during long-term heat stress acclimation. However, mRNA expression of IL2 and IL6 genes in peripheral blood was higher in mid-winter than summer for all of three age groups of tropical and temperate goat breeds suggesting an essential role of IL2 and IL6 genes in modulation of cold stress by maintaining cellular homeostasis (Maurya et al. 2013).

HSP70 is a molecular chaperone that plays a crucial role in biological process of protein. It was found to be the most temperature sensitive gene correlated with thermotolerance (Park et al. 2007; Singh et al. 2017). The role of this gene was demonstrated in goats by the expression analysis to be effective in cold adapted than in heat-adapted breeds in both summer and winter (Banerjee et al. 2014). This role and expression of HSP70 was different among breeds of sheep in arid and semi-arid areas and more expressed in winter (Singh et al. 2017). Moreover, the relative expression of HSP70 gene in skin of Tharparkar and Karen fares cattle during different seasons were high in summer and winter than in spring, indicating that this gene works under adverse cold or hot thermal stress.

## Conclusion

Barki sheep are well adapted to harsh desert environment. They have high tolerance capacity to withstand heat stress by altering several physiological mechanisms such as increasing ST, WT, PR and RR while maintaining normal blood plasma constituents. Also, IL-2, IL-6 and HSP 70 are temperature sensitive genes. Their expressions could be used as molecular marker for heat/cold stress.

## Data Availability

Available upon request.

## Code availability

Available upon request.
